# Observations on Some Rock Carvings of Surgical Instruments in East Africa

**Published:** 1945

**Authors:** M. Nierenstein

**Affiliations:** Formerly Reader in Bio-Chemistry, University of Bristol


### OBSERVATIONS ON SOME ROCK CARVINGS OF SURGICAL INSTRUMENTS IN EAST AFRICA

BY

M. NIERENSTEIN, D.Sc.

*Formerly Reader in Bio-Chemistry, University of Bristol.*

LONG before I had an inkling that I would ever visit Africa, I was interested in the ruins of Zimbabwe[1] and other works[2] bordering on the subject. My reading, however was purely amateurish as I have no knowledge whatever of archæology, mineralogy, etc., the essentials for the serious study of the subject. When I finally did visit Africa, my interests were botanical with a strong surgical bias, as I had intended to specialize in surgery when I studied medicine nearly 50 years ago. As soon as the opportunity offered itself I visited the Great Zimbabwe[3] and I shall always remember the awe I felt when I stood before this edifice of a forgotten civilization. Subsequent visits to Africa took me many times to Zimbabwe and some of the other Zimbabvic ruins so widely scattered all over Rhodesia, as evident from the admirable map published by Hall and Neal[1]; and on one occasion I spent over two weeks botanizing at Zimbabwe and its surroundings in a 60 miles radius. Although my time was devoted to botany, my thoughts turned to that eternal question : Who were the builders of these ruins ?

In this essay I give my observations on drawings showing instruments traced by me in Southern and Northern Rhodesia, the Eastern corner of the Belgian Congo, Tanganyika, Portuguese East Africa and Nyasaland. Of the 160 illustrations I have seen 18 were on rocks and hills, 48 on stones on inland roads and paths and 94 were situated on river banks, lakes and in swamps. This is contrary to the usual distributions of rock carvings and paintings which are generally found on the tops of hills, mountains and rocks.

Here again we are faced with the question who were the men who cut these figures and why were they cut ? Lack of knowledge suggests the hypothesis that these illustrations indicate the dwelling places of practising surgeons. These practising surgeons were not of the type of the traditional witch doctor (Shopo) but trained Inyatas[4], and the difference between a witch doctor and an inyata is fully realized by the East African native.

This brings us to the second question : where had these inyatas been trained ? At Zimbabwe, for which I found no evidence, at Alexandria or elsewhere ? To judge from the illustrations I have seen, these surgeons were in no way under the influence of Arabic surgery. Arabic surgical instruments are angular[5] and thus easy to draw, but none of the illustrations I have seen showed any evidence of angularity. Zimbabwe surgery, to give it a provisional name, was an Egypto-Greco-African product, and for the time being East African surgery is best dealt with separately from other types of surgery.

The illustrations found by me consisted of :—11 cupping cups, one of which was a cow's horn as still used by the Basuto, 58 knives, 3 knives for the Caesarean operation, 9 small bows and arrows for the puncturing of blood vessels, 8 squares with holes in the middle, 17 trepanning instruments, 4 instruments for the operation on haemorrhoids according to Hippocrates, 27 surgical needles, 17 pincettes, 13 canulas, 7 nails with flattened heads, 2 illustrations showing the Hippocrates operation for haemorrhoids and 1 illustration showing the urinary system of a eunuch. The absence of instruments for amputations is noteworthy and is probably due to the natives' dislike of losing limbs, a dislike which has survived to the present day.

The following observations are essential for the interpretation of the surgical instruments used by the Zimbabwe surgeons :—

1. The squares with holes were at first difficult to understand, but my visits to the swamp of Lukanga and the lakes of St. Joseph and Rukwa in the company of Inkos Inkota[6] offered a solution. I noticed that some of the carriers wore thin squares of lead strapped on their backs and that the abscesses they had were visible through holes in these strips. In the evenings the carriers used to unstrap and foment the abscesses with a mash of hot leaves and then next morning strap the lead strips on. By taking swabs which contained lead, I found that the pus dissolved the lead. From this I concluded that the lead acts as a disinfectant of the wound. The Inkos also told me that this method of treating festering wounds, especially war wounds, was very old and he showed me old assagai scars which had been treated in this manner 30 or more years ago.

2. Gurlt[5] points out that the caesarean operation is of very long standing and was already practised in Egypt during the XI and XII Dynasties (2130–1930 B.C.) perhaps earlier.

3. *Trepanning* was used and is still practised in East Africa in mental diseases such as epilepsy, hysteria, etc. Reference should be made to Larry[7], Schreger[8], Tillmanns and Hartmann[10] as to the theoretical aspect of this form of treatment.

4. The following is a free translation describing the operation for haemorrhoids according to Hippocrates (460–337 B.C.): "I recommend an iron rod 8–10 inches long and of the thickness of a little finger hammered out at one end to the size of a small obolas (silver 3d. piece). On the day preceding the operation the patient is given a strong purgative. On the day of the operation the patient is laid on his back and a hard pillow made from wood covered with hide is pushed half way down the spine. The haemorrhoids are exposed by pressure with one's fingers on the anus. At the same time the instrument is made red hot and each individual haemorrhoid touched with red hot plate. Care must be taken that not a single haemorrhoid is missed: this is not impossible as each haemorrhoid is the size of a small berry. The blood oozes out as the hot iron touches the haemorrhoids and care must be taken that it does. During the operation, the hands, legs and head of the patient are held down; he must be kept stationary. Yelling of the patient is useful as it forces the rectum out and thus exposes otherwise hidden haemorrhoids." In the two pictures found by me the patient is not on his back, but his backside faces the onlooker and the instrument touches the anus.

5. The nails with flattened heads were used, according to Godard[11, 12] in castration. Godard had the opportunity of seeing a number of castrated boys and of examining them. He reports that long nails with the flattened side outwards were inserted in the bladder through the wound after removal of the scrotum. This kept the wound open and facilitated urine excretion through the open hole during which time urination could only take place when sitting down, as the penis is atrophied for a considerable time. During this time which lasts from 8–10 months the urine contains pus. As the wound heals the penis recovers and becomes more or less normal, but the patient suffers from suppression of urine and has to insert a canula and to take strychnine. The illustration seen by me showed the penis and canula. Of interest is also that the face of the individual depicted was that of a Semite.

It is obviously impossible to judge from the drawings the material from which the instruments were made, but some were, to judge from the illustrations, made out of stone. Again, whereas the illustrations given by Gurlt[5] had been made from the actual instruments found in museums and had thus either wooden or leather handles, as for example the knives used in the caesarean operations, the carvings I have seen had no handles. There were several illustrations of small stone knives which are still used in East Africa for circumcision, clitoridectomy and in operations for calculus.

During a prolonged stay on the Limpopo, I witnessed the preparations for circumcision and clitoridectomy. From 2 to 4 a.m. the boys and girls, some 40 in number of each sex, stood up to their waists in the cold water of the Limpopo which numbs the lower part of their bodies, and soon after 4 o'clock they were operated on. During all that time several hundred natives swam round them and protected them against crocodiles, which abound in the Limpopo.

I was later on shown the operating knives, made from broken pieces of bottle glass, perfectly ground and sharpened. I was given one of these knives, and for a long time, until I lost it, it kept its sharp edge.

To judge from the surgical illustrations I have seen, the " Zimbabwe " surgery had its roots in the Stone Age and, using that admirable work of Hall and Neal, I have come to the conclusion that it stopped at about A.D. 600, that is before the time East Africa got into contact with the Arabs.

### REFERENCES AND ANNOTATIONS.

[1] Bent, *The Ruined Cities of Mashonaland*, 1892, London. Peters, *The Eldorado of the Ancients*, 1902, London. Hall and Neal, *The Ancient Ruins of Rhodesia*, 1904, London. Johnson, *British Central Africa . . .* , 2 vols., 1919, London.

[2] Sollas, *Ancient Hunters . . .* , 1915, London.

[3] Zimba means lion in several native dialects.

[4] Inyata means snake, wisdom, namely " doctor."

[5] Gurlt, *Geschichte der Chirurgie . . .* , vol. i,, 1898, Berlin. I have used Gurlts' plates i, ii and iii for the identification of the surgical instruments here described. Compare also Walsh, *Mediaeval Medicine*, 1920, London.

[6] Nierenstein, *Pharm. Journ.*, 1943, vol. 150, page 121.

[7] Larry, *Relation historique et chirurgical de l'expedition de l'armée d'Orient en Egypte et en Syrie*, 1803, Paris.

[8] Schreger, *Chirurgische Versuche*, 2 vols., 1811, 1822, Nürnberg.

[9] Tillmanns, *Arch. f. Klin Chirurgie*, 1883, vol. 28, page 775.

[10] Hartmann, *Naturgeschl.-medicinische Skizzen der Nilländer*, 1887, Berlin.

[11] Godard, *Egypte et Palestine, observations médicales et scientifiques*, 1867, Paris.

[12] According to Burckhard, *Travels in Nubia*, 1819, London, Zawyet-et-deyer near Suit in Upper Egypt produced as many as 200 eunuchs every year from Negroes, Europeans, etc. The ages of the boys castrated varied from eight to twelve years and they were sold for £100 to £200 per head when they had reached the age of eighteen years.

